# Effect of Non-alcoholic Fatty Liver Disease on the Risk of Synchronous Liver Metastasis: Analysis of 451 Consecutive Patients of Newly Diagnosed Colorectal Cancer

**DOI:** 10.3389/fonc.2020.00251

**Published:** 2020-02-28

**Authors:** Yan Lv, Hai-jun Zhang

**Affiliations:** Department of Oncology, The Affiliated Zhongda Hospital of Southeast University, Medical School of Southeast University, Nanjing, China

**Keywords:** non-alcoholic fatty liver disease, colorectal cancer, synchronous liver metastasis, cirrhosis, fibrosis-4 index, non-alcoholic fatty liver disease fibrosis score

## Abstract

**Background:** The purpose of this study was to investigate the effect of non-alcoholic fatty liver disease (NAFLD) on the risk of synchronous colorectal liver metastasis (synCRLM).

**Methods:** A retrospective analysis was performed on 451 consecutive patients with newly diagnosed colorectal cancer (CRC) from January 2014 to January 2019. According to the presence of NAFLD, the CRC patients were divided into two groups, NAFLD group (60 cases) and the control group (391 cases). The clinicopathological features and the prevalence of synCRLM between the two groups were compared. Logistic regression analysis was used to analyze the risk factors of synCRLM. Different non-invasive liver fibrosis scoring models were used to evaluate the effect of advanced fibrosis and cirrhosis stage in NAFLD on the prevalence of synCRLM.

**Results:** The prevalence of synCRLM was significantly higher in patients with NAFLD than that in patients without NAFLD (18.33 vs. 7.42%; χ^2^ = 7.669, *P* = 0.006). A logistic regression analysis indicated that NAFLD, CEA, CA19-9, and lymph node status were risk factors for synCRLM, and NAFLD showed the highest hazard ratio (3.930 [95% confidence interval: 1.616 ~ 9.560]). In NAFLD patients, both fibrosis-4 index (FIB-4) and NAFLD fibrosis score (NFS) were significantly lower in those with synCRLM compared to those without synCRLM [FIB-4: 1.246 (0.833 ~ 1.276) vs. 1.436 (1.016 ~ 2.699), *Z* = −2.130, *P* = 0.033; NFS: −1.282 (−2.407 ~ −0.262) vs. −0.255 (−1.582 ~ 0.755), *Z* = −2.302, *P* = 0.021; Mann-Whitney test].

**Conclusion:** NAFLD may be associated with increased liver metastasis, and for NAFLD-related advanced liver fibrosis and cirrhosis may be associated with reduced synchronous liver metastasis in CRC patients. However, the correlation between simple steatosis and steatohepatitis remains to be further determined. Certain factors such as NAFLD, lymph node metastasis, elevated levels of preoperative CEA and CA19-9 are suggesting a high risk of synCRLM.

## Introduction

Colorectal cancer (CRC) is one of the most well-established malignancies of the digestive system, which is also the third most common malignant tumor in the world and the fourth most frequent cause of cancer-related deaths. It is estimated that by 2030, this disease burden will increase by nearly 60% (1). There will be 2.2 million new CRC patients and 1.1 million deaths from CRC (2). Distant metastasis is the most important independent risk factors for poor prognosis in CRC patients. The liver is the most common site for distant metastasis of CRC and is often the only organ involved (3). Previous studies have reported that more than 50% of CRC patients will develop liver metastasis in the process of the disease, about 14–25% of CRC patients are found to have synchronous liver metastasis at the time of diagnosis, and about 10–25% of CRC patients develop metachronous liver metastasis during follow-up after the initiation of treatment. Approximately 80–90% of CRC patients with liver metastasis could not initially get radical resection of liver metastases, and these people who unable to get radical resection have a very low 5-year survival rate (4–8). Therefore, whether liver metastasis occurs or not is the crucial point affecting the prognosis of CRC, and understanding the risk factors of liver metastasis may be one of the effective strategies to reduce the circumstance of liver metastasis.

At present, with the change of lifestyle and dietary structure, the prevalence of insulin resistance and obesity is gradually increasing, and the incidence of adult non-alcoholic fatty liver disease (NAFLD) is moderately increasing, which has become one of the most recurrent causes of chronic liver diseases across the globe. The worldwide prevalence of NAFLD is ~25.24% (9). NAFLD is a chronic and persistent pathological process from hepatic steatosis to obvious hepatic injury. It includes two pathological diagnoses with different prognosis: non-alcoholic fatty liver (NAFL) and non-alcoholic steatohepatitis (NASH) (10). Current studies have shown that NAFL is a disease with or without progression of histopathological changes very slowly over time. NAFL is a relatively benign stage for NAFLD which is easy to be reversed, roughly 10–20% of NAFL can be converted into NASH (11, 12). NASH has a potentially invasive progression process that can lead the way to liver fibrosis, cirrhosis, hepatocellular carcinoma and even induce distant organs carcinogenesis, such as CRC (13, 14).

Most studies have reported that NAFLD is an independent risk factor for the development of CRC (15, 16), while the effect of NAFLD on colorectal liver metastasis (CRLM) remains unclear. Some studies have outlined that NAFLD reduced the risk of CRLM (17–20), while others suggested the opposite conclusion (21–23). Updated studies that further investigate the mechanism underlying the effects of NAFLD on CRLM are certainly needed. Referring to the well-known “seed-soil” hypothesis in the mechanism of tumor metastasis (24, 25). Herein, CRC cells as “seed” and the liver with NAFLD as “soil” have been taken into consideration to explore whether “soil,” the liver with NAFLD is responsible for metastasis. Our strict definition of “synchronous” ruled out the effect of variations in the follow-up treatment on the likelihood of liver metastasis. Meanwhile, we further analyzed the effects of different stages of NAFLD on synchronous colorectal liver metastasis (synCRLM), which is the first known study to evaluate the impact of the dynamic progression of NAFLD on synCRLM. In this retrospective clinical study, we obtained unique and interesting results and proposed new insights into the association between NAFLD and synCRLM.

## Materials and Methods

### Study Population

We conducted a retrospective study of all newly diagnosed CRC patients who were continuously admitted to the Affiliated Zhongda Hospital of Southeast University between January 2014 and January 2019.

The inclusion criteria were as follows: (1) all patients confirmed as CRC histopathologically, (2) did not receive any surgery, interventional treatment, chemotherapy or radiotherapy in other hospitals before admission, (3) for the purpose of detecting the presence of NAFLD and distant organ metastasis, all patients screened by preoperative chest, abdominal and pelvic regions imaging, such as ultrasound, computed tomography (CT) and magnetic resonance imaging (MRI), (4) complete medical records.

The exclusion criteria were as follows: (1) CRC patients with synchronous distant metastases except for liver, (2) CRC patients accompanied by malignant tumors in other parts of the body, (3) CRC patients with liver lesions caused by alcohol, viruses, drugs, genetics and other pathogenic factors. In total, 451 patients were included in this study.

The study was approved from an ethics approval by the Ethics Committee of Zhongda Hospital, Southeast University. The study protocol protected the private information of enrolled patients in accordance with the provisions of the Helsinki Declaration.

### Data Characteristics

Data collection included demographic information [gender, age, height, weight, body mass index (BMI)] and clinical data, including the following: NAFLD, hypertension, diabetes or impaired fasting glucose (IFG), hepatitis B surface antigen (HBsAg), aspartate aminotransferase (AST), alanine aminotransferase (ALT), glutamyl transferase (GGT), alkaline phosphatase (ALP), total bilirubin (TBIL), direct bilirubin (DBIL), indirect bilirubin (IBIL), triglyceride (TG), platelet (PLT), albumin (ALB) and the following tumor markers: carcinoembryonic antigen (CEA), carbohydrate antigen 19-9 (CA19-9) and alpha-fetoprotein (AFP). Tumor characteristic which based on the guideline of the American Joint Committee on Cancer staging, with detailed descriptions on each patient's pathology report, including primary tumor site, tumor size, tumor type (protuberant, ulcerative, infiltrative), differentiation (well, moderate, poor), tumor (T) status, lymph node (N) status, TNM stage, vascular invasion, nerve invasion, and KRAS, NRAS, BRAF mutation status. The original data for all patients in this study were shown in Supplementary Data Sheet 1.

### Imaging Evaluation of the Liver, Metastasis Assessment

Compared with liver biopsy as the gold standard, liver imaging examination is more commonly used in clinical work. Ultrasound, CT and MRI have high sensitivity and specificity in the diagnosis of liver steatosis (26, 27). According to the Chinese guidelines for the prevention and treatment of NAFLD (2018 updated edition) (28), the diagnosis of NAFLD should meet the following requirements: (1) liver biopsy indicates significant hepatic steatosis and/or imaging examination, such as ultrasound, CT and MRI results consistent with the manifestations of fatty liver, which liver fat content is more than 5%, (2) no history of alcohol consumption more than 30 g for men and 20 g for women per day, and (3) rule out other factors or diseases that contribute to hepatic steatosis.

Based on the imaging examination results, patients were divided into the NAFLD group and control (without NAFLD) group. The baseline clinicopathological parameters and prevalence of synCRLM were compared between the two groups. SynCRLM was defined as the synchronous or prior diagnosis of liver metastasis together with primary CRC. All diagnoses of liver metastasis were confirmed by pathological biopsy or independently confirmed by CT or MRI by two senior radiologists.

### Calculation of Non-invasive Liver Fibrosis Scoring Models

NAFLD fibrosis score (NFS), Fibrosis-4 Index (FIB-4), AST-to-PLT ratio index (APRI), and BRAD score were calculated and used as the indicators of advanced liver fibrosis or cirrhosis. These scores are calculated as follows:

$$\begin{array}{l} NFS=-1.675+0.037\times age\;\left(years\right)+0.094\times BMI\;\left(\frac{Kg}{m^{2}}\right)\\ \;+\;1.13\times IFG\;or\;diabetes(yes=1,no=0)\\ \;+\;0.99\times (AST÷ALT)-0.013\times PLT\;\left(\frac{10^{9}}{L}\right)\\ \;-\;0.66\times ALB\;(\frac{g}{dl}) \end{array}$$

$$\begin{array}{l} FIB-4=age\;\left(years\right)\times AST\left(\frac{IU}{L}\right)÷[PLT\;\left(\frac{10^{9}}{L}\right)\\ \;\times \sqrt{ALT\left(\frac{IU}{L}\right)}] \end{array}$$

$$\begin{array}{l} APRI=AST\left(\frac{IU}{L}\right)÷upper\;limit\;of\;the\;liver\;biopsy\;of\;AST\\ \;\times 100÷PLT\left(\frac{10^{9}}{L}\right) \end{array}$$

and the upper limit of normal of AST in our hospital is 37IU/L. BRAD score combines the three variables into one weight sum (BMI ≥28 = 1 point, AST/ALT ratio ≥0.8 = 2 points, diabetes = 1 point), which can be used to predict advanced fibrosis quickly and easily.

As reported previously, those patients with NFS higher than 0.676, FIB-4 higher than 1.30, APRI higher than 0.50 and the BRAD score of 2–4 were diagnosed as having an advanced fibrotic liver (29–33), patients in NAFLD group were grouped according to high-level (>0.676) or low-level (≤0.676) NFS, high-level (>1.30) or low-level (≤1.30) FIB-4, high-level (>0.50), or low-level (≤0.50) APRI and high-score (2–4 score) or low-score (0–1 score) BRAD. NFS, FIB-4, APRI, and BARD score were compared between the NAFLD patients with or without synCRLM.

### Statistical Methods

The correlations between demographics, clinical and pathological data of the two groups were analyzed. Categorical variables were compared by χ^2^ test, correction χ^2^ test or Fisher's exact test. Numerical variables conforming to normal distribution were compared using student *t* test, comparisons for numerical variables with skewed distribution were performed using the Mann-Whitney *U* test. Significant risk factors for synCRLM were analyzed first by univariate logistic regression analysis and then by multivariate logistic regression analysis. All the statistical tests considered two-sided *P* value <0.05 as statistically significant. Statistical analysis was performed using SPSS version 25.0 software (IBM Corporation, Armonk, NY, USA).

## Results

### Baseline Parameters of CRC Patients

**Figure 1:**
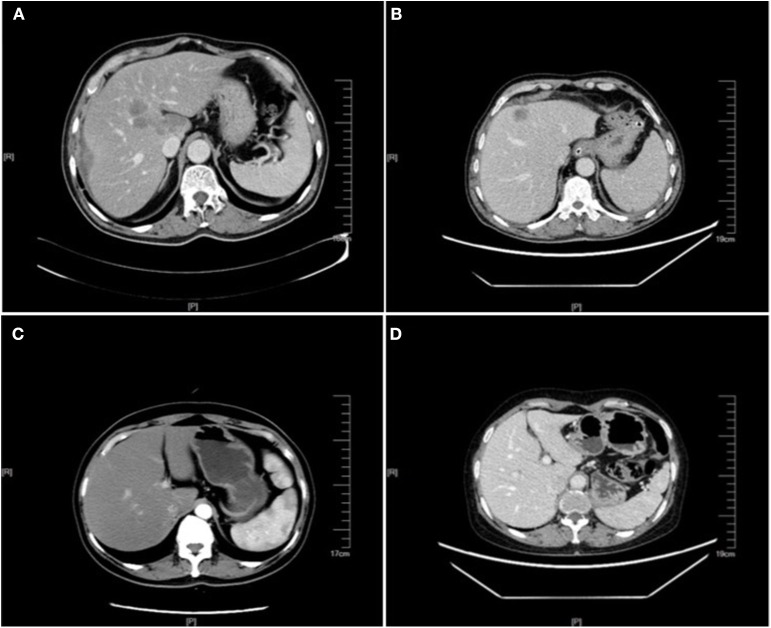
Enhanced CT images of liver metastasis, NAFLD and normal liver in the CRC patients. **(A)** NAFLD group, synCRLM^+^: The shape of the liver was regular, and the proportion of each lobe was within the normal range. The density of the liver parenchyma was lower than that of the spleen in each stage of enhancement, and multiple round-like enhancement shadows could be seen in the liver parenchyma with unclear boundary. **(B)** Control group (without NAFLD), synCRLM^+^: A patchy abnormal enhancement foci with unclear boundary was seen in the left inner lobe of the liver, showing slight enhancement, while no abnormal density or enhancement was observed in the remaining liver parenchyma. **(C)** NAFLD group, synCRLM^−^: The density of liver parenchyma decreased, which was lower than that of the spleen in the same layer. **(D)** Control group (without NAFLD), synCRLM^−^: No abnormal density and enhancement foci were found in the liver parenchyma.

**Figure 2:**
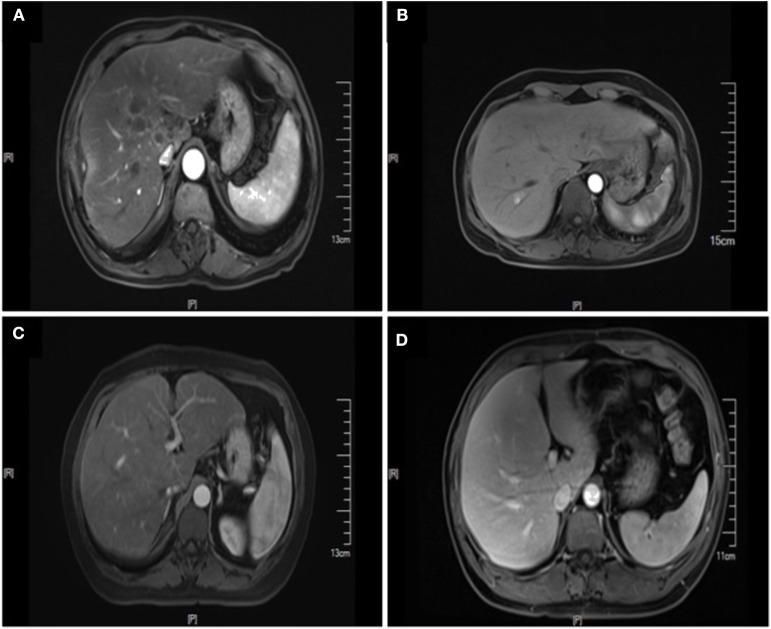
Enhanced MRI images of liver metastasis, NAFLD and normal liver in the CRC patients. **(A)** NAFLD group, synCRLM^+^: The density of liver parenchyma decreased significantly in the reverse position. Multiple patchy abnormal signal shadows were seen in the liver, especially in the hepatic hilum. Necrosis was observed in the center of some lesions, with low enhancement in the center of the enhanced lesions and line-like enhancement at the edges. **(B)** Control group (without NAFLD), synCRLM^+^: Enhanced scan showed a round-like long T1 and long T2 signal shadow near the right hepatic vein in the right posterior lobe of the liver. In the arterial phase, the lesion was significantly enhanced, and the surrounding areas could be seen with enhanced flocculent perfusion. **(C)** NAFLD group, synCRLM^−^: The density of liver parenchyma decreased significantly in the reverse position. **(D)** Control group (without NAFLD), synCRLM^−^: The liver was in regular shape, and the proportion of each lobe was harmonious. No obvious abnormal signal shadow was observed in the liver parenchyma.

**Figure 3:**
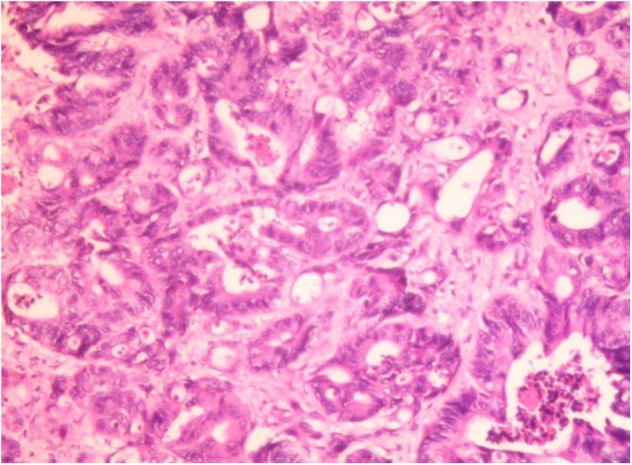
Hepatic histopathology of a case of CRC patient. Accumulation of fat vacuoles (steatosis) and abnormal tumor cells were observed in hepatic lobules. Immunohistochemical result: CK7 (focal +), CK20 (–), CDX2 (+), villin (+), Ki67 (about 60%+), SATB2 (+), CDH17 (+). The liver metastasis was consistent with the origin of colorectal adenocarcinoma.

A total of 451 patients were confirmed for the analysis during the study period. Among them, 60 (13.30%) patients were diagnosed with NAFLD, and 391 (86.70%) patients were regarded as the control group. The baseline clinicopathological parameters of the two groups are presented in Table 1. The weight and BMI of the NAFLD patients were significantly higher than that of the control patients (weight: *P* = 0.022; BMI: *P* < 0.001). NAFLD was found at a higher incidence in patients with diabetes or IFG (41.67 vs. 19.69%, *P* < 0.001). There were no significant differences in the sex, age, height, hypertension, HBsAg, primary tumor site, tumor size, tumor type, tumor differentiation, T status, LN status, vascular invasion, nerve invasion, and KRAS, NRAS, BRAF mutation status. The prevalence of synCRLM was 18.33% (11/60) in the NAFLD group, which was significantly higher than the prevalence of 7.42% (29/391) in the control group (χ^2^ = 7.669, *P* = 0.006). The overall primary disease stage (TNM) was different between the two groups (χ^2^ = 7.939, *P* = 0.047), but there was no significant difference between the two groups during stage I to III (χ^2^ = 0.267, *P* = 0.862), while there was a significant difference between stage I~III and IV (χ^2^ = 7.669, *P* = 0.006), which was attributed to the difference in distant metastasis (M) status between the two groups. Figures 1, 2 showed enhanced CT and enhanced MRI images of liver metastasis, NAFLD and normal liver in CRC patients in this study, respectively. Figure 3 showed the histopathological manifestation of resection of liver metastasis in a CRC patient in this study.

**Table 1:** Clinicopathological parameters of primary colorectal cancer in the NAFLD group and control group.

**Factor**	**NAFLD group (*n* = 60)**	**Control group (*n* = 391)**	***χ^2^/t/Z* Value**	***P***
SynCRLM (yes/no)	11/49	29/362	7.669	0.006
Sex (male/female)	30/30	236/155	2.307	0.129
Age (year)	66 (45 ~ 93)	66 (29 ~ 94)	−0.040	0.968
Height (cm)	165 (150 ~ 182)	165 (140 ~ 188)	−0.655	0.513
Weight (Kg)	66.5 (38.0 ~ 99.0)	63.0 (36.0 ~ 100.0)	−2.296	0.022
BMI (Kg/m^2^)	24.71 ± 3.74	23.12 ± 2.98	−3.703	<0.001
Hypertension (yes/no)	33/27	193/198	0.662	0.416
Diabetes or IFG (yes/no)	25/35	77/314	14.351	<0.001
HBsAg (positive/negative)	2/58	28/363	0.688	0.407
**Primary CRC**				
Tumor site			1.682	0.431
Left-sided colon	14 (23.33)	99 (25.32)		
Right-sided colon	28 (46.67)	149 (38.11)		
Rectum	18 (30.00)	143 (36.57)		
Tumor type			2.655	0.264
Protuberant	18 (30.00)	153 (39.13)		
Ulcerative	39 (65.00)	227 (58.06)		
Infiltrative	3 (5.00)	11 (2.81)		
Tumor size (≥5/<5, cm)	31/29	193/198	0.111	0.739
Differentiation			0.365	0.546
Well and moderate	53 (88.33)	355 (90.79)		
Poor	7 (11.67)	36 (9.21)		
T status			0.412	0.521
T1–T2	9 (15.00)	72 (18.41)		
T3–T4	51 (85.00)	319 (81.59)		
LN status			1.582	0.208
N0	27 (45.00)	210 (53.71)		
N1–N2	33 (55.00)	181 (46.29)		
Stage of disease (TNM)			7.939	0.047
Stage I	7 (11.67)	62 (15.86)		
Stage II	19 (31.67)	141 (36.06)		
Stage III	23 (38.33)	159 (40.66)		
Stage IV	11 (18.33)	29 (7.42)		
Vascular invasion (yes/no)	19/41	128/263	0.027	0.869
Nerve invasion (yes/no)	18/42	94/297	0.990	0.320
KRAS mutation status			6.465	0.039
Mutation	11 (18.33)	89 (22.76)	0.977	0.323
No mutation	7 (11.67)	93 (23.79)		
Unknown	42 (70.00)	209 (53.45)		
NRAS mutation status			5.356	0.067
Mutation	0 (0.00)	6 (1.53%)		
No mutation	18 (25.53)	17 (44.76)		
Unknown	42 (74.47)	210 (53.71)		
BRAF mutation status			3.584	0.151
Mutation	1 (1.67)	7 (1.79)		
No mutation	20 (33.33)	180 (46.04)		
Unknown	39 (65.00)	204 (52.17)		

*Data are presented as No. (%) unless otherwise indicated. The data of age, height, weight is presented as median (percentile min ~ percentile max). The data of BMI is presented as mean ± standard deviation*.

*NAFLD, non-alcoholic fatty liver disease; synCRLM, synchronous colorectal liver metastasis; BMI, body mass index; IFG, impaired fasting glucose; CRC, colorectal cancer; HBsAg, hepatitis B surface antigen*.

### Risk Factors for synCRLM

**Figure 4:**
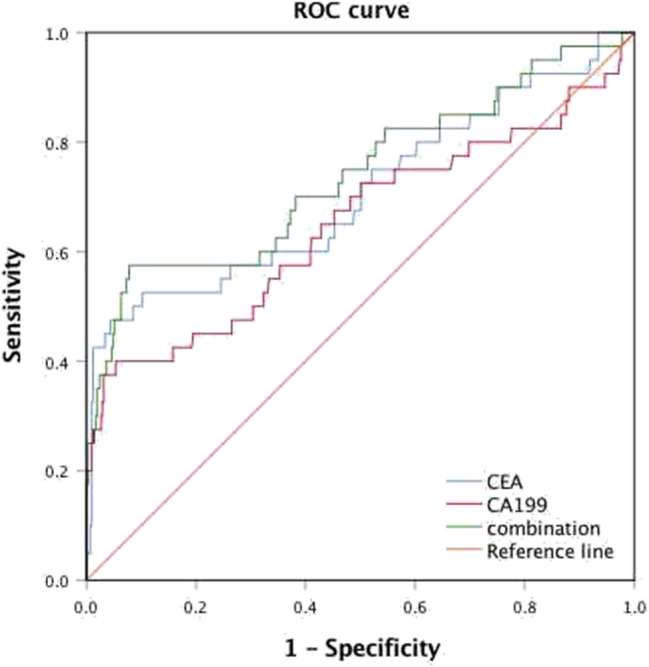
Receiver operating characteristic (ROC) analysis for the prediction of synchronous liver metastasis in colorectal cancer patients.

Among all patients, 40 CRC patients were diagnosed with synchronous liver metastasis, and the total prevalence of synCRLM was 8.87%. According to whether the occurrence of synCRLM, they were divided into synCRLM^+^ and synCRLM^−^ group. Chi-square test, independent sample *t* test and non-parametric test were performed on the clinicopathological parameters of the two groups, and the results showed that there were no significant differences in HBsAg, AFP, ALT, AST, ALP, GGT, TBIL, DBIL, IBIL, ALB, TG, PLT, tumor size, tumor type, nerve invasion, and NRAS, BRAF mutation status between the two groups (*P* > 0.05) (Supplementary Table 1). Following univariate logistic regression analysis, CEA, CA19-9, primary tumor site, differentiation, T status, LN status, vascular invasion and NAFLD were chosen for the subsequent multivariate logistic regression analysis. As the loss of KRAS mutation status was more than 50%, it was not included in the multivariate logistic regression analysis, which finally indicated that NAFLD was considered an independent risk factor for liver metastasis and NAFLD had the highest hazard ratio (3.930[95% confidence interval: 1.616 ~ 9.560]). Moreover, advanced LN status (N1 and N2), elevated levels of preoperative CEA and CA19-9 were linked with increased risks of synCRLM (Table 2). The sensitivity of CEA and CA19-9 in predicting synCRLM were 47.5 and 40.0%, respectively; the specificities were 95.6 and 94.6%, respectively. ROC curve analysis displayed that the optimal critical value of CEA was 34.86 ng/mL and CA19-9 was 53.82 U/mL, which suggested that preoperative CEA levels above 34.86 ng/mL and preoperative CA19-9 levels above 53.82 U/mL were associated with greater risks of synCRLM. The sensitivity and specificity of the combined diagnosis of CEA and CA19-9 for synCRLM were 57.5 and 92.2%, respectively. The combination of CEA and CA19-9 (Area under curve (AUC) [95%CI], 0.739 [0.642~0.835]) exhibited a better predictive value than CEA (AUC [95%CI], 0.705 [0.603~0.807]) or CA19-9 (AUC [95%CI], 0.649 [0.541~0.835]) alone (Figure 4).

**Table 2:** Univariate and multivariate logistic regression analysis of the significant predictors for synchronous colorectal liver metastasis.

**Variables**	**Univariate analysis**		**Multivariate analysis**	
	**OR (95% CI)**	***P* value**	**OR (95% CI)**	***P* value**
NAFLD	2.802 (1.317 ~ 5.965)	0.008	3.930 (1.616 ~ 9.560)	0.003
CEA	1.009 (1.004 ~ 1.013)	<0.001	1.005 (1.002 ~ 1.008)	0.003
CA19-9	1.016 (1.010 ~ 1.023)	<0.001	1.013 (1.006 ~ 1.020)	<0.001
**PRIMARY TUMOR SITE**				
Colon	1		1	
Rectum	0.422 (0.189 ~ 0.938)	0.034	0.485 (0.184 ~ 1.280)	0.485
**DIFFERENTIATION**				
Well and moderate	1		1	
Poor	6.838 (3.235 ~ 14.455)	<0.001	2.520 (0.956 ~ 6.642)	0.062
**T Status**				
T1–T2	1		1	
T3–T4	9.426 (1.276 ~ 69.642)	0.028	2.293 (0.283 ~ 18.578)	0.437
**LN STATUS**				
N0	1		1	
N1–N2	5.033 (2.264 ~ 11.188)	<0.001	2.955 (1.014 ~ 8.612)	0.047
Vascular invasion	3.948 (2.012 ~ 7.748)	<0.001	1.660 (0.669 ~ 4.117)	0.274

*NAFLD, Non-alcoholic fatty liver disease; CEA, carcinoembryonic antigen; CA19-9, carbohydrate antigen 19-9; OR, odds ratio; CI, confidence interval*.

### The Influence of Advanced Liver Fibrosis and Cirrhosis on synCRLM

Sixty patients with NAFLD were divided into two groups according to whether synchronous liver metastasis occurred. The median (25–75th percentile) NFS and FIB-4 were −1.282 (−2.407 ~ 0.262) and 1.246 (0.833 ~ 1.276) in those with synCRLM, both were significantly lower compared to the median of −0.255 (−1.582 ~ 0.755) and 1.436 (1.016 ~ 2.699) in those without synCRLM (NFS: *Z* = −2.302, *P* = 0.021, Mann-Whitney test; FIB-4: *Z* = −2.130, *P* = 0.033, Mann-Whitney test). There was no significant difference in APRI between the two groups (*Z* = −1.939, *P* = 0.052, Mann-Whitney test). FIB-4, APRI, NFS, and BRAD score of NAFLD patients were grouped according to the recommended threshold values, respectively, to compare the prevalence of synCRLM in each group at different levels (Table 3). These results demonstrated that the risk of synCRLM in NAFLD patients with advanced fibrosis/cirrhosis was significantly lower than patients without advanced fibrosis/cirrhosis.

**Table 3:** Comparison of synCRLM between different levels of FIB-4, APRI, NFS, and BRAD score.

**Group**	**With synCRLM**	**Without synCRLM**	***χ^2^* value**	***P***
FIB-4			5.455	0.020
High-level FIB-4	2 (6.67%)	28 (93.33%)		
Low-level FIB-4	28 (93.33%)	21 (70.00%)		
APRI			0.198	0.656
High-level APRI	1 (9.09%)	10 (90.91%)		
Low-level APRI	10 (20.41%)	39 (79.59%)		
NFS			3.901	0.048
High-level NFS	1 (4.17%)	23 (95.83%)		
Low-level NFS	10 (27.78%)	26 (72.22%)		
BRAD score			0.174	0.677
High-score BRAD	8 (16.33%)	41 (83.67%)		
low-score BRAD	3 (27.27%)	8 (72.73%)		

*synCRLM, synchronous colorectal liver metastasis; FIB-4, Fibrosis-4 Index; APRI, aspartate aminotransferase-to-platelet ratio index; NFS, non-alcoholic fatter liver disease fibrosis score*.

## Discussion

In our study, we found that NAFLD may be associated with increased synCRLM. However, NAFLD is a general term for multiple disease stages, including NAFL, NASH, NAFLD-related fibrosis, cirrhosis and even hepatocellular carcinoma. We need to analyze the different effects that may occur at different stages in the progression of NAFLD. We observed that when NAFLD progressed to advanced fibrosis or cirrhosis were associated with decreased synCRLM. Meanwhile, we used multivariate analysis to suggest that NAFLD, CEA, CA19-9, and LN status were associated with an increased incidence of synchronous liver metastasis in CRC patients.

The “seed-soil” hypothesis suggests that metastatic tumor cells will migrate to an area where the local microenvironment is favorable. Most prior studies have focused on determining how “seeds” (cancer cells) promote metastasis, while ignoring the role of “soil” (target organs). The preferential growth of metastasis in an organ is due to its unique biological characteristics and its endogenous microenvironment containing special cells and molecular components that promote the formation of metastasis. Interventions that target blocking these signals may be effective in inhibiting metastasis. Our study aimed at the “soil” factor, re-recognized the impact of NAFLD on synCRLM and highlighted the role of liver microenvironment changes in the formation of liver metastases.

It is notable that the phenomenon of liver metastasis is one of the main aspects determining the prognosis of CRC patients. We need to pay more attention to the moment when liver metastasis occurs. Synchronous liver metastasis may represent worse biological behavior of the primary tumor and even worse prognosis. Although with the continuous deepening of the medical research and the rapid development of medical technology, the diagnosis and treatments of CRC are becoming more and more standardized, but often because of a variety of reasons, including differences of medical services levels in different regions, limitations of patients' family conditions, differences of patients' individual physical conditions and compliance, etc., there is still a significant imbalance in the treatment of CRC. At the same time, the standard of diagnosis and treatments of CRC are constantly upgrading. Even if the guidelines are strictly followed, the standard treatments received by patients in different periods are not alike. CRC patients who receive curative resection will have a longer expected survival time and a longer follow-up time. While, the disparity in the treatment among CRC patients, there is no reasonable explanation for the difference in the incidence of metachronous liver metastasis, which is not conducive to truly understanding the impact of NAFLD on CRLM. Based on the above situation, it is bound to affect the final outcomes if the synchronous and metachronous liver metastasis are not strictly distinguished. In our study, the prevalence of synCRLM was 8.87%. However, previous clinical studies have reported that the prevalence of synCRLM is about 14–25% (4–8). The decreased prevalence of synCRLM in our study may be related to inconsistencies in the definition of “synchronous.” Previous studies have defined synchronous as the development of a liver metastasis within 6 months and even up to 12 months after the primary CRC has been diagnosed (34). In addition, in recent years, the general population has gradually deepened their understanding of CRC, as well as the progress of early screening and diagnosis technology of CRC. Therefore, the decrease in the incidence of liver metastasis is within the expected range. However, our study defined as liver metastasis detected concurrently or before the primary CRC with a strict definition of synchronous can exclude the effect of subsequent treatments on liver metastasis and reflect the differences of cancer biology more accurately, providing guidance for individualized treatment.

In this study, the prevalence of NAFLD was 13.30%, and the median prevalence of NAFLD in China was 15.3% (11.3–24.6%), which was in line with the prevalence trend of NAFLD in China (35). Based on previous studies, there was an independent interrelation between NAFLD and both diabetes or IFG, and this relationship was closely related to BMI changes (36, 37), which was consistent with this observed trend in our study. Compared with the control group, CRC patients in NAFLD group had a significantly higher rate of synchronous liver metastasis. Further multivariate logistic regression analysis proposed that NALFD was associated with an increased incidence of synchronous liver metastasis.

A meta-analysis included a total of 10,349 CRC patients from 10 studies, the results demonstrated that patients with chronic liver diseases including hepatitis, cirrhosis, and fatty liver had significantly lower incidences of CRLM than those with the normal livers conditions (OR = 0.32, 95% CI 0.26–0.38, *P* < 0.001). However, in this meta-analysis, synchronous and metachronous liver metastasis were included. Only 3 studies explored the CRLM correlation with fatty liver, and the correlation with NAFL or NASH was not further studied in detail (17). A study explored the relationship between hepatic steatosis (HS) with the incidence of CRLM by objective quantifying steatosis using a CT scan. Patients with liver-spleen attenuation ratio lower than 1.1 were objectively defined as HS, and the results manifested that the incidence of synchronous metastasis in liver observed in HS patients was lower than that in normal liver patients, but there was no statistically significant difference (3.2 vs. 9.6%, *P* = 0.06) (18). Some retrospective studies suggested that liver metastasis of CRC was less frequently detected in patients with fatty liver, both of these studies did not distinguish between synchronous and metachronous metastasis and were reported a long time ago, whereas fatty liver was diagnosed only by two-dimensional ultrasound (19, 20). These prior studies underestimated the risk of NAFLD for liver metastasis. However, recent studies reported contradictory results that NAFLD patients have a higher risk of liver metastasis and postoperative recurrence of liver metastasis when compared with non-NAFLD patients, although these studies were looking at metachronous liver metastasis (21–23).

Some animal research also presented a different conclusion. The murine models revealed that hepatic steatosis induced by a high-fat diet increased the metastatic tumor burden of colon cancer tumors metastatic to the liver, and the changes in the liver microenvironment caused by fatty liver established a more sensitive microenvironment for metastasis when compared with normal liver (38–40). However, the opposite conclusion was discovered in a rat model (41). This difference might be attributed to the application of various experimental conditions, such as different tumor cell lines, atypical types of mice, unlike injection sites and methods, etc. And it was still necessary to pay attention to the different effects of the etiology and degree of hepatic steatosis on the results.

To farther explore the disparities in the prevalence of synCRLM in a different stage of NAFLD, we compared FIB-4, APRI, NFS, and BRAD score in NALFD patients with and without synCRLM. Compared with liver biopsy, FIB-4, APRI, NFS, and BRAD score may have moderate diagnostic efficacy for advanced fibrosis or cirrhosis, but these scoring models are non-invasive, inexpensive, widely available, and the dynamic change process of liver fibrosis can be understood (29–33). NFS and FIB-4 scoring were most accurately determined severe liver diseases compared with APRI and BRAD scoring (42). Our results demonstrated that both FIB-4 and NFS scoring in NAFLD patients without synCRLM were significantly higher than that in NAFLD patients with synCRLM. Meanwhile, the prevalence of synCRLM was lower in the high-level FIB-4 and the high-level NFS group than in the low-level FIB-4 and the low-level NFS group. APRI and BARD score did not reach the statistical significance. Therefore, the possible explanation for the above results is that advanced fibrosis or cirrhosis may decrease the risk of synCRLM.

Indeed, earlier studies had recommended that cirrhosis reduces the risk of liver metastasis (43, 44). To be specific, our study observed that when NAFLD progressed to advanced fibrosis or cirrhosis, it may be associated with a decrease in synchronous liver metastasis. We consider several hypotheses that might explain this phenomenon: (1) It may be related to morphological changes in the liver. Gradually accumulation of triglycerides in the liver leads to the compression and deformation of intrahepatic blood vessels. With the aggravation of the severity of the disease, when it develops to advanced fibrosis or cirrhosis, leading to the distortion and reconstruction of intrahepatic blood vessels. Especially in cirrhosis, scar tissue and regenerative nodules are formed, which directly compress the blood vessels and shunt the hepatic artery and portal vein into the central vein, thereby affecting the blood supply in the liver, making it difficult for tumor cells to enter the liver and not conducive to the growth of metastatic tumors. (2) Cirrhosis is associated with increased intrahepatic portal vein resistance. With the increasing severity of the degree of cirrhosis, peak venous velocity decreases significantly, and the contraflow of a portal venous system can be observed in patients with cirrhosis. The disruption of venous shunting on portal blood flow may prevent tumor cells from reaching the liver (45). (3) Kupffer cells in cirrhosis are activated to release pro-inflammatory factors, which further promote fibrogenesis and up-regulate the expression of Fas receptor, thus increasing the sensitivity of metastatic tumor cells to FasR-mediated apoptosis, and then being killed and cleared by tumor-infiltrating cells (46). (4) With the aggravation of fibrosis, the expression of metalloproteinase tissue inhibitors and other anti-proteinases in hepatocytes and hepatic stellate cells increased, making the overall activity of metalloproteinase reduced, providing resistance to metastasis (47).

Currently, there is no effective non-invasive scoring model to predict the occurrence of NAFL and NASH. In the progression of NAFLD, NAFL plays a dominant role. About 10–20% of NAFL can develop into NASH, and about 20% of NASH can further progress into hepatic fibrosis. Progressive accumulation of fibrosis can lead to the formation of advanced fibrosis or cirrhosis (11–14). Consequently, the proportion of advanced fibrosis and cirrhosis in NAFLD is very low. Based on our findings, we hypothesize that simple steatosis and steatohepatitis might be associated with an increased risk of synchronous liver metastasis. Inflammation renders a key role in tumor progression and contributes to the formation of metastases (48). Obesity is closely related to the development of NAFLD, which is a state of chronic low-grade inflammation. Many cytokines and growth factors synthesized and released by adipocytes have direct carcinogenic effects in the gastrointestinal tract, elevated levels of free fatty acids can induce inflammatory pathways (49). The increase of insulin-like growth factor-I (IGF-I) level can enhance the proliferation and anti-apoptosis effect of tumor cells. IGF-I deficient mice developed smaller primary tumors and reduced the burden of liver metastasis (50). Adiponectin and leptin generally affect cellular behavior in opposite ways. Hypoadiponectinemia is a characteristic feature of NASH and is not linked with insulin resistance (51). Overexpression of adiponectin leads to reduced metastasis, adiponectin can inhibit tumor angiogenesis and down-regulate invasive signaling pathways. Leptin expression elevated significantly with the increase in obesity. Leptin receptors are overexpressed in CRC, and leptin has been shown to reduce the apoptosis of CRC cells and promote tumor growth and metastasis (52). Tumor-related macrophages (TAMs) are a major part of the tumor microenvironment that promotes tumor growth, and NOD-like receptor C4 (NLRC4) is a component of the inflammatory body complex. A recent study further confirmed that a high-fat diet-induced NAFLD promotes the growth of CRLM. More importantly, this study explored the role of NLRC4 and interleukin (IL)-1β in the growth of metastatic liver tumors under NAFLD-related liver microenvironment, emphasizing the effect of inflammation changes in NAFLD on the metastatic microenvironment. The study concluded that in NAFLD, NLRC4 contributes to M2 TAMs polarization, IL-1β and vascular endothelial growth factor (VEGF) production in TAMs, which promotes the growth of metastatic liver tumors in CRC (40).

To be obvious, the present study has several limitations. First, this was a single-center study, and there may be some inevitable selection biases. At the same time, this study was retrospective, so only the association between NAFLD and liver metastasis could be determined, rather than the cause-effect relationship. In addition, the sample size of this study was limited and needs to be verified by prospective studies with a large sample size in multiple centers. Second, all non-invasive liver fibrosis scoring models involved in this study were based on the assessment of common clinical parameters and functional alternations in the liver, these alternations may not accurately reflect the replacement of extracellular matrix or changes in fibroblastic cells and cannot fully assess change in the liver microenvironment. Pathological findings can provide strong evidence of immunology and histology. Third, the effect of simple steatosis and steatohepatitis on the prevalence of synchronous liver metastasis was calculated by the “sum of local effects” method, without any actual data or model support.

Despite these limitations, this study keeps the center of attention on the synchronous liver metastasis which can more accurately reflect the role of the “soil” factor in the mechanism and formation of metastasis, also re-recognize the impact of NAFLD on the microenvironment of metastasis. As a matter of fact, this is the first study to explore the specific effects of NAFLD-related fibrosis/cirrhosis on the development of synCRLM. The results of this clinical research provide evidence for the effect of NAFLD on synchronous liver metastasis in CRC patients and potential explanations for different stages in NAFLD. Future studies need to further verify the effects of simple steatosis and steatohepatitis on synchronous liver metastasis, and more prospective clinical trials and animal model experiments are needed to verify and explore the potential mechanism of the interrelation with liver metastasis. As the incidence of NAFLD is increasing rapidly worldwide, follow-up clinical trials of CRC patients should pay more attention to the prevalence of NAFLD in different populations. The interpretation of whether patients with NAFLD and the extent of liver lesions can help to determine the risk of synCRLM and provide guidance for subsequent treatment.

## Conclusion

NAFLD may be associated with increased synchronous liver metastasis in CRC patients, while NAFLD progresses to advanced fibrosis or cirrhosis may be associated with decreased synCRLM. This study provides a sufficient and referable clinical basis for exploring the role of NAFLD-related liver microenvironment changes in tumor metastasis.

## Data Availability Statement

All datasets generated for this study are included in the article/Supplementary Material.

## Author Contributions

YL completed data collection, data analysis, and manuscript drafting. HZ directed the arrangement of the study and supervised the whole writing of the manuscript and manuscript revision. Both the authors conceived the review and approved of the final analysis and results.

### Conflict of Interest

The authors declare that the research was conducted in the absence of any commercial or financial relationships that could be construed as a potential conflict of interest.
