# Supplementary material for: Effect of Non-alcoholic Fatty Liver Disease on the Risk of Synchronous Liver Metastasis: Analysis of 451 Consecutive Patients of Newly Diagnosed Colorectal Cancer
Source: Front Oncol. 2020 Feb 28;10:251. doi: 10.3389/fonc.2020.00251 (PMC7059642; doi:10.3389/fonc.2020.00251)
Supplement: Supplementary file 1 [file Table_1.docx]

Supplementary Material

**Supplementary Table 1. Comparison of clinicopathological parameters between the synCRLM^+^ group and synCRLM^-^ group.**

| Factor | synCRLM^+^ group (n=40) | synCRLM^-^ group (n=411) | *χ^2^/t/Z* Value | *P* Value |
| --- | --- | --- | --- | --- |
| NAFLD (yes/no) | 11/29 | 49/362 | 7.669 | 0.006 |
| CEA (ng/mL) |  |  | 6.214 | 0.013 |
| >5 | 24（60.00） | 163（39.66） |  |  |
| ≤5 | 16（40.00） | 248（60.34） |  |  |
| CA19-9 (U/mL) |  |  | 28.095 | <0.001 |
| >39 | 16（40.00） | 39（9.49） |  |  |
| ≤39 | 24（60.00） | 372（90.51） |  |  |
| AFP (ng/mL) |  |  | / | 0.527 |
| >7 | 1（2.50） | 7（1.70） |  |  |
| ≤7 | 39（97.50） | 404（98.30） |  |  |
| ALT (IU/L) |  |  | <0.001 | 0.995 |
| >40 | 1（2.50） | 16（3.80） |  |  |
| ≤40 | 39（97.50） | 395（96.11） |  |  |
| AST (IU/L) |  |  | 0.049 | 0.826 |
| >35 | 1（2.50） | 19（4.62） |  |  |
| ≤35 | 39（97.50） | 392（95.38） |  |  |
| ALP (IU/L) |  |  | 1.167 | 0.280 |
| >135 | 3（7.50） | 12（2.92） |  |  |
| ≤135 | 37（92.50） | 399（97.08） |  |  |
| GGT (IU/L) |  |  | 3.818 | 0.051 |
| >45 | 9（22.50） | 44（10.71） |  |  |
| ≤45 | 31（77.50） | 367（89.29） |  |  |
| TBIL (umol/L) |  |  | 0.233 | 0.637 |
| >20.0 | 2（5.00） | 35（8.52） |  |  |
| ≤20.0 | 38（95.00） | 376（91.48） |  |  |
| DBIL (umol/L) |  |  | 0.747 | 0.388 |
| >6.0 | 5（12.50） | 30（7.30） |  |  |
| ≤6.0 | 35（87.50） | 381（92.70） |  |  |
| IBIL (umol/L) |  |  | 0.025 | 0.875 |
| >18.0 | 2（5.00） | 13（3.16） |  |  |
| ≤18.0 | 38（95.00） | 398（96.84） |  |  |
| ALB (g/L) |  |  | 0.112 | 0.738 |
| ≥40 | 17（42.50） | 186（45.26） |  |  |
| <40 | 23（57.50） | 225（54.74） |  |  |
| TG (mmol/L) |  |  | 0.699 | 0.403 |
| ≥1.70 | 10（25.00） | 80（19.46） |  |  |
| <1.70 | 30（75.00） | 331（80.54） |  |  |
| PLT (10^9^/L) |  |  | 0.007 | 0.935 |
| ≥125 | 39（97.50） | 394（95.86） |  |  |
| <125 | 1（2.50） | 17（4.14） |  |  |
| BMI (Kg/m^2^) | 23.44（16.41-31.33） | 22.96（14.79-32.87） | -0.522 | 0.601 |
| Hypertension (yes/no) | 21/19 | 205/206 | 0.100 | 0.752 |
| Diabetes or IFG (yes/no) | 7/33 | 95/316 | 0.657 | 0.418 |
| HBsAg (positive/negative) | 1/39 | 29/382 | 0.595 | 0.440 |
| Primary CRC |  |  |  |  |
| Tumor site |  |  | 4.712 | 0.030 |
| Colon | 32（80.00） | 258（62.77） |  |  |
| Rectum | 8（20.00） | 153（37.23） |  |  |
| Tumor type |  |  | 0.206 | 0.900 |
| Protuberant | 14（35.00） | 157（38.20） |  |  |
| Ulcerative | 25（62.50） | 227（58.64） |  |  |
| Infiltrative | 1（2.50） | 13（3.16） |  |  |
| Tumor size (≥5/<5, cm) | 25/15 | 199/212 | 2.891 | 0.089 |
| Differentiation |  |  | 23.997 | <0.001 |
| Well and moderate | 27（78.95） | 381（92.70） |  |  |
| Poor | 13（21.05） | 30（7.30） |  |  |
| T status |  |  | 7.120 | 0.008 |
| T1-T2 | 1（2.50） | 80（19.46） |  |  |
| T3-T4 | 39（97.50） | 331（80.54） |  |  |
| LN status |  |  | 21.343 | <0.001 |
| N0 | 7（17.50） | 229（55.72） |  |  |
| N1-N2 | 33（82.50） | 182 （44.28） |  |  |
| Vascular invasion (yes/no) | 25/15 | 122/289 | 17.868 | <0.001 |
| Nerve invasion (yes/no) | 14/26 | 98/313 | 2.430 | 0.119 |
| KRAS mutation status |  |  | 10.515 | 0.005 |
| Mutation | 15（37.50） | 85（20.68） | 10.865 | 0.001 |
| No mutation | 2（5.00） | 98（23.85） |  |  |
| Missing data | 23（57.50） | 228（55.47） |  |  |
| NRAS mutation status |  |  | 0.873 | 0.643 |
| Mutation | 1（2.50） | 6（1.46） |  |  |
| No mutation | 16（40.00） | 176（42.82） |  |  |
| Missing data | 23（57.50） | 229（55.72） |  |  |
| BRAF mutation status |  |  | 0.247 | 0.938 |
| Mutation | 0（0.00） | 8（1.95） |  |  |
| No mutation | 19（47.50） | 181（44.04） |  |  |
| Missing data | 21（52.50） | 222（54.01） |  |  |

Data are presented as No. (%) unless otherwise indicated. BMI: median (percentile 25-percentile 75).

Abbreviations: synCRLM, synchronous colorectal liver metastasis; CEA, carcinoembryonic antigen; CA19-9, carbohydrate antigen 19-9; AFP, alpha fetoprotein; ALT, alanine aminotransferase; ALP, alkaline phosphatase; AST, alanine aminotransferase; GGT, glutamyl transferase; TBIL, total bilirubin; DBIL, direct bilirubin; IBIL, indirect bilirubin; ALB, albumin; TG, triglyceride; PLT, platelet; BMI, body mass index; IFG, impaired fasting glucose; CRC, colorectal cancer; NAFLD, non-alcoholic fatty liver disease; HBsAg, hepatitis B surface antigen.
